# Depression and anxiety symptoms among women in post-conflict Somalia: a cross-sectional study in maternal and child health centres

**DOI:** 10.3389/fgwh.2025.1652133

**Published:** 2026-02-16

**Authors:** Gallad D. Hassan, Juweria N. L. Abshir, Fatumo Osman

**Affiliations:** 1 Faculty of Graduate Studies and Research, Somali National University, Mogadishu, Somalia; 2 School of Health and Welfare, Dalarna University, Falun, Sweden

**Keywords:** anxiety, depression, mental health, post-conflict, sense of coherence, social support, women

## Abstract

**Objective:**

Mental health issues can be regarded as a severe public health problem that affects low-, middle-, and high-income countries worldwide. However, certain populations in post-conflict countries may have particularly vulnerable mental health, such as women of childbearing age in Somalia. Despite our understanding of this vulnerable group, data on mental health among women living in conflict and post-conflict settings are still scarce. Therefore, this study aims to identify the prevalence of depressive and anxiety symptoms among women living in post-conflict Somalia and their association with sense of coherence (SOC), perceived social support, and individual sociodemographic factors.

**Methods:**

Data were collected from 900 women who attended nine maternal and child health centres (MCHs) in the Banadir region of Somalia, using a self-report validated questionnaire in Somali. This questionnaire was based on the depression component of the Patient Health Questionnaire-9, the Generalised Anxiety Disorder scale, SOC, and sociodemographic factors. The data were analysed using multiple logistic regression with STATA software (v. 16) to determine the factors associated with depression and anxiety.

**Results:**

Depressive symptoms (36%) and anxiety symptoms (38%) were mainly associated with higher income, unstable housing, and comorbid anxiety or depression. Sense of coherence consistently showed a protective effect. No significant associations were found with pregnancy status, MCH visits, social support, or most other sociodemographic factors.

**Conclusions:**

The study identified a high prevalence of depressive and anxiety symptoms among women attending MCH centres, with low socioeconomic status and low sense of coherence emerging as key associated factors. These findings underscore the urgent need to integrate routine mental health screening and support services within primary health care and MCH centres to improve early identification, reduce stigma, and strengthen women's well-being in post-conflict Somalia.

## 1 Introduction

Mental health issues cause severe public health problems that affect low-, middle-, and high-income countries worldwide. More specifically, common mental disorders (CMDs) have been suggested to be the most frequent disorders affecting individuals globally. These CMDs are regarded as nonpsychotic mental health problems, such as depressive, anxiety, somatoform, and adjustment disorders that compromise daily functioning (1, 2). More interestingly, when shifting the focus to low- and middle-income countries, CMDs such as depressive and anxiety disorders become highly prevalent. For example, Khai (3) revealed that the prevalence of CMDs in these countries is much higher because of a plethora of socioenvironmental issues, including poverty, food insecurity, overcrowding, and various diseases, such as malaria and human immunodeficiency virus, alongside a lack of proper medical care.

Within this context, the occurrence of depressive and anxiety disorders is significantly greater among women of childbearing age compared to any other demographic group (4). Alongside the aforementioned socioenvironmental issues, other factors may explain the prevalence of depressive and anxiety disorders among women. From a sociocultural perspective, Bezerra et al. (1) note that this prevalence can be explained by sociodemographic factors related to the female gender, such as domestic violence and stressors associated with women's multiple social roles. Furthermore, from a biological perspective, women are vulnerable to experiencing depression and anxiety during their childbearing years, partly due to hormonal fluctuations that affect mood regulation. Changes in estrogen during phases such as the premenstrual, postpartum, and menopausal periods are associated with increased emotional sensitivity which may place women at a greater biological risk of developing depressive and anxiety disorders (1). Some studies have highlighted the association between CMDs and different sociodemographic characteristics, such as living in poverty (4), being unmarried, being a housewife (1), and being a victim of domestic violence (5). Much is still unknown about the association between CMDs and sociodemographic factors; therefore, more research is imperative, as CMDs among women, predominantly depressive and anxiety disorders, have consequential implications. For example, CMDs can contribute to an increased risk of suicide, substance abuse, and poor physical health (4) and negative economic implications for women, such as unemployment and financial insecurity (1).

To combat these CMDs and minimise their related risks, women should employ effective coping mechanisms to maintain and improve their mental and emotional well-being (6, 7). For example, Aaron Antonovsky advanced the concept of salutogenesis, which posits that life experiences shape the sense of coherence (SOC) that helps to mobilise resources to cope with stressors and manage tension successfully (30). Similarly, Pachi et al. (8) examined sense of coherence (SOC) to understand its role as a coping mechanism in relation to CMDs and how individuals may navigate stressors such as illness, remain resilient in the face of environmental challenges, and maintain both their physical and their emotional well-being. SOC-13 comprises three interrelated components: sense of comprehensibility, sense of manageability, and sense of meaningfulness. These elements guide individuals in organising stimuli, addressing demands, and finding value in acting against stressors in their environment (9).

Previous studies have found that the SOC scale significantly influences women's perceptions of their health. The scale is based on individuals' ability to maintain health and manage stress, with SOC scores reflecting their capacity to remain healthy, even during stressful conditions (10). In particular, a strong SOC is linked to a higher quality of life and reduced intensity of depressive and anxiety symptoms in patients with various psychiatric disorders (9–12). Furthermore, it is well established that SOC is often inversely related to depressive and anxiety symptoms, with even mild levels of depression being associated with lower levels of SOC (9, 13).

Regarding Somalia, years of conflicts and natural disasters have left its population in a vulnerable state, and the country is facing a shortage of healthcare professionals, especially in the mental health field (14). With women of childbearing age being the most vulnerable to CMDs, such as depressive and anxiety disorders, it is essential to study their depressive and anxiety symptoms in the under researched setting of post-conflict countries, in addition to the role of SOC in this setting. Moreover, in Somalia, stigma and cultural barriers prevent individuals from seeking help. Mental health care at the community level throughout Somalia, whether integrated into primary health-care services or provided independently, is practically absent. Therefore, many people depend on their families and communities for support (14). This reliance on family and community support often falls short because of the pervasive stigma surrounding mental health, leading many individuals to avoid seeking help altogether (14, 15). However, seeking family and community support is the only option available when health-care services are limited.

Despite growing awareness of the vulnerability of women in conflict and post-conflict environments, empirical evidence on CMDs such as anxiety and depression among Somali women remains scarce. Prolonged displacement, insecurity, economic hardship, and limited access to mental health services create conditions where mental health problems are likely widespread, yet these settings remain understudied. Furthermore, most existing research in Somalia is qualitative or focuses on general populations [e.g., (16)], leaving women of childbearing age largely unexamined. Considering the lack of prior research, it is essential to build a solid knowledge base by examining the prevalence of depressive and anxiety symptoms among Somali women and to explore how sociodemographic factors, social support, and SOC influence mental health. Therefore, this study aims to identify the prevalence of depressive and anxiety symptoms among Somali women living in post-conflict Somalia and their association with SOC, perceived social support, and individual sociodemographic factors. The expected contribution of this work is to inform policy, guide intervention development, and strengthen the evidence base for improving women's mental health in post-conflict Somalia.

## 2 Methods

### 2.1 Design and settings

A descriptive cross-sectional design was used in this study, which was conducted in Mogadishu, the capital city of Somalia, which has been affected by prolonged civil war and conflict over the last three decades. Mogadishu is now recovering from this long conflict and hosts most of the internally displaced persons (IDP) camps in Somalia. In this study, nine main maternal and child health centres (MCHs) were selected based on the population density of the nine Mogadishu districts, as per an unpublished document from the Banadir Regional Administration. Of these nine districts, three had high population density, three had low population density, and three had an average population density. All selected MCHs were funded by government and nongovernmental organisations to provide primary health care, including antenatal care, treatment of malnutrition, immunisation, basic laboratory diagnosis, family planning, and health education services, among others. Approximately 80 to 200 women visit these MCHs daily and are served by between 18 and 48 health-care professionals.

### 2.2 Population and data collection

The study population comprised women who attended the nine selected MCH facilities during the study period. Each of the nine districts contributed a quota of 100 women, resulting in a total sample of 900 participants. A convenience sampling approach was used. In each district, recruitment was conducted at the primary Maternal and Child Health (MCH) facility, which is supported by both governmental and non-governmental organizations. The data were collected between August and September 2021 by four experienced data collectors, supervised by one of the first authors (GD). The data collectors received refreshment training on data collection specifically for this study, which was conducted by the authors (GD, FO), who also supervised their subsequent data collection activities. The questionnaire was available in Somali; however, as most participating women could not read or write in Somali, the data collectors read the questions aloud and recorded the participants' responses.

Ethical approval was obtained from the School of Public Health at Somali National University (SNU/SPHR/005/2021). Permission was sought from the district and health facility levels before contacting the MCHs. Information about the study was given to the health-care professionals working at each MCH. Together with one of the first authors (GD), the data collectors visited each MCH. The women visiting the MCHs were informed about the study individually. Those who expressed interest in participating and provided verbal consent were included in the study. In total, 900 women provided consent and completed the questionnaire with the help of the data collectors. One participant who did not complete the questionnaire was excluded from the analysis; thus, 899 women were included. All data were collected anonymously.

### 2.3 Instruments

The data were collected through a self-report validated questionnaire in Somali. The dependent variables were the Patient Health Questionnaire-9 (PHQ-9) and the seven-item version of the Generalised Anxiety Disorder scale (GAD-7). The independent variables included the 13-item version of the SOC scale (SOC-13) and questions on sociodemographic factors, including age, number of children, marital status, pregnancy status, regular visits to MCHs, educational status, employment status, housing situation, and monthly household income.

All instruments were available in Somali except SOC-13, which we translated back and forth between English and Somali using the World Health Organisation guidelines for the Process of translation and adaptation of instruments (17).

Before data collection, the questionnaire was tested at another MCH that was not included among the MCHs selected for this study.

#### 2.3.1 Patient health questionnaire-9

The PHQ-9 is a nine-item self-report questionnaire assessing depressive symptoms and disorders (18). Individuals reported their depressive symptoms over the past 2 weeks on a 4-point Likert scale (0 = not at all, 1 = several days, 2 = more than half the days, and 3 = nearly every day). The Somali version of the PHQ-9 has been validated with strong internal consistency, homogeneity, and reliability (19). The severity of depression was classified according to the total score (mild, 5–9; moderate, 10–14; and severe, 15–27).

#### 2.3.2 Generalised anxiety disorder scale

The GAD-7 scale is a seven-item self-report questionnaire that measures anxiety symptoms and their severity. Individuals described their anxiety symptoms over the past 2 weeks based on a four-point Likert-type scale (0 = not at all, 1 = several days, 2 = >1 week, and 3 = nearly every day). The severity of anxiety was classified by the total score (mild, 5–9; moderate, 10–14; and severe, 15–21) (20). The GAD-7 scale was used in a previous study with a Somali population and demonstrated good internal consistency and reliability (21).

#### 2.3.3 Sense of coherence scale

The SOC-13 scale is a 13-item self-report questionnaire that measures coping with adversity using a 7-point Likert Scale. Five items (i.e., items 1, 2, 3, 7, and 10) are reversed in scoring, and the total scores range between 13 and 91. To the best of our knowledge, the SOC-13 scale has not been validated in Somali. However, the scale has shown good internal consistency in different settings, with Cronbach's *α* values ranging from 0.75 to 0.91 across different studies (20, 21).

### 2.4 Data analysis

**Figure 1:**
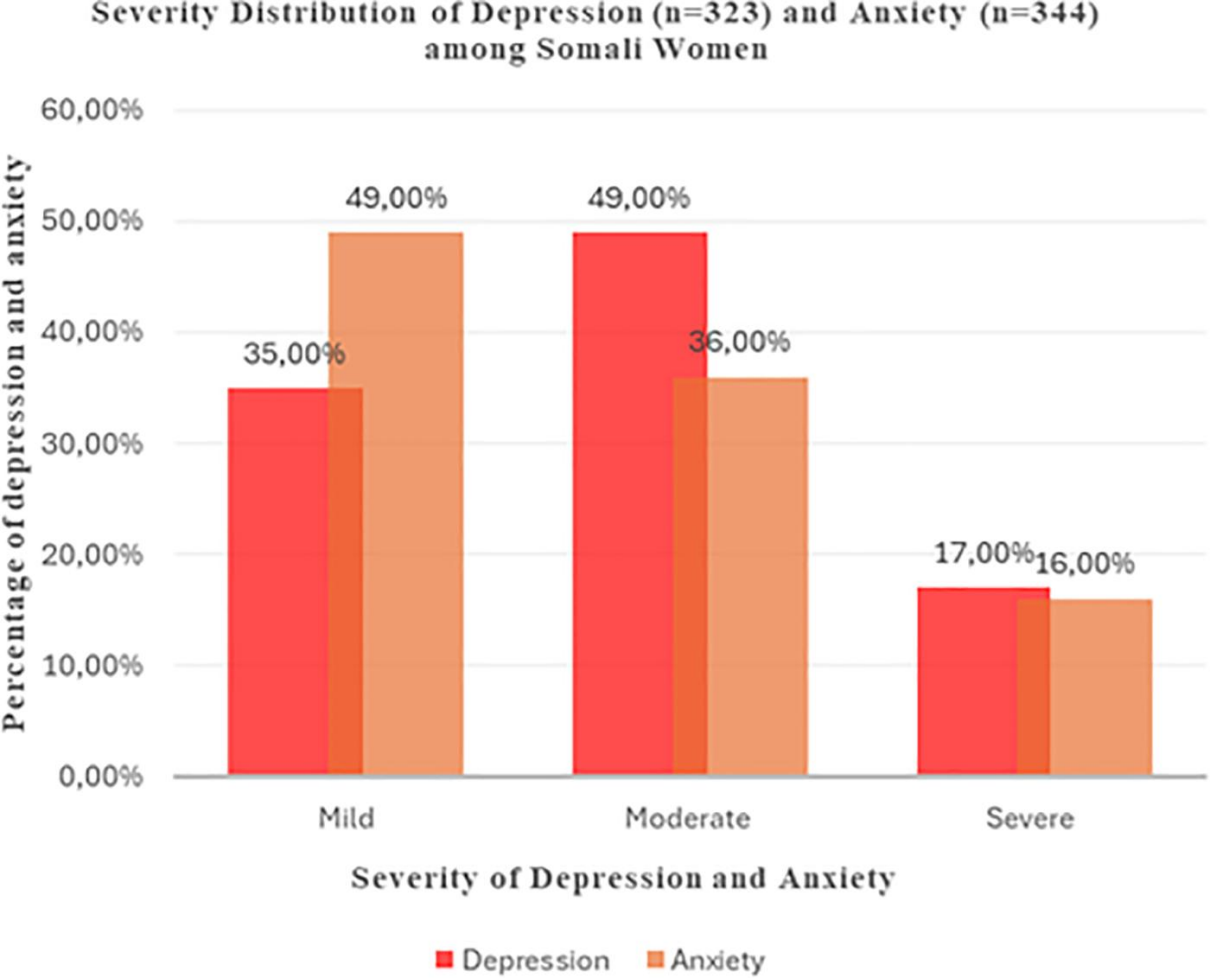
Prevalence of depressive and anxiety symptoms among Somali women (*n* = 899).

Descriptive statistics, including means, standard deviations (SD), ranges, frequencies, and proportions, were computed using STATA software (v.16; StataCorp LLC, College Station, TX, USA). Multicollinearity among the independent variables was assessed prior to the regression analysis, and no evidence of multicollinearity was detected. Because the outcome variables (PHQ-9 and GAD-7) showed right-skewed distributions, robust standard errors were applied to improve the reliability of the regression estimates. The prevalence of depressive and anxiety symptoms is illustrated in Figure 1.

Both univariate and multivariate linear regression analyses were conducted to examine the associations between the dependent variables (PHQ-9 and GAD-7) and the independent variables (SOC-13, perceived social support, and sociodemographic factors). Regression coefficients (*β*) and their corresponding 95% confidence intervals (CIs) are reported. Statistical significance was defined as a *p*-value < 0.05.

## 3 Results

The participants' average age was 28 years (SD ± 6.55). Approximately one-third (35%) of the participants were pregnant, and the vast majority (95%) were married. On average, the women had five children (SD ± 3). Regarding their educational attainment, the majority of participants (85%) had received informal education (that is, they attended only a Koran school), and 94% were unemployed (not in paid employment). Regarding their household income, only 9.5% had a monthly household income of ≥200 US dollars; 50% lived in rented houses, and 14% lived in IDP camps. Most participants (80%) reported feeling supported by their families, friends, and neighbourhoods. Their mean scores for depression, anxiety, and SOC were 4.72 (SD ± 5.15), 4.79 (SD ± 4.87), and 59.81 (SD ± 9.34), respectively (see Table 1).

**Table 1:** Participants’ characteristics (*N* = 899).

Variables	*n* (%)
Age, mean (SD)	28.09 (6.55)
Number of children, mean (SD)	5 (3)
Pregnancy	
Pregnant	318 (35%)
Not pregnant	581 (65%)
Regularly visit MCHs^a^	
No	114 (13%)
Yes	785 (87%)
Marital status	
Married	855 (95%)
Single	44 (5%)
Educational level	
Formal education	135 (15%)
Informal education	764 (85%)
Employment	
Employed	58 (6%)
Unemployed	841 (94%)
Household type	
Owner	58 (6%)
Renter	453 (50%)
Relatives’ house	266 (30%)
IDP camps	122 (14%)
Household income (USD)	
<100	373 (41%)
100–199	442 (49%)
200–299	80 (9%)
>300	4 (0.5%)
Perceived social support	
No	180 (20%)
Yes	719 (80%)
PHQ-9 total score, M (SD)^b^	4.72 (5.15)
GAD-7 total score, M (SD)^c^	4.80 (4.87)
SOC-13 total score, M (SD)^d^	59.81 (9.34)

a Maternal and child health centers;

b Patient Health Questionnaire-9 (Depression);

c General Anxiety Disorder-7 scale (Anxiety);

d 13-item Sense of Coherence scale.

### 3.1 Prevalence of depressive and anxiety symptoms among Somali women

Table 1 shows that the mean levels for the PHQ-9 and GAD-7 scales were 4.72 (SD ± 5.15) and 4.79 (SD ± 4.87), respectively, which indicates the low mean levels of depressive and anxiety symptoms among Somali women in Somalia. When these symptoms were classified into mild, moderate and severe, however, the results showed that 323 (36%) and 344 (38%) women reported depressive and anxiety symptoms, respectively.

Among the 323 women with depressive symptoms, 114 (35%) had mild symptoms, 153 (49%) had moderate symptoms, and 56 (17%) had severe symptoms. Similarly, among the 344 women who had anxiety symptoms, 168 (49%) had mild symptoms, 122 (36%) had moderate symptoms, and 54 (16%) had severe symptoms (see Figure 1).

### 3.2 Associations between depressive symptoms (PHQ-9), sense of coherence, social support, and sociodemographic factors

Univariate regression analyses showed that several sociodemographic factors were positively associated with depression scores (Table 2). The number of children was associated with a 0.242 increase in depression score (*β* = 0.242, 95% CI: 0.125 to 0.358). Being single was associated with a higher depression score (*β* = 2.155, 95% CI: 0.638 to 3.670) compared to married participants.

**Table 2:** Univariate and multivariate linear regression analyses for depression (PHQ-9), SOC, social support, and demographic factors. (*n* = 899).

Variables	Categories	Univariate *β* (95% CI)	Multivariate *β* (95% CI)
Age (Year)		0.05 (0.0008–0.099)*	−0.011 (−0.052 to 0.03)
Number of children		0.242 (0.125 to 0.358)***	0.056 (−0.036 to 0.148)
Marital status	Married	Ref.	Ref.
	Single	2.155 (0.638 to 3.67)**	0.087 (−0.697 to 0.873)
Pregnancy status	Yes	Ref.	Ref.
	No	−.57 (−1.285 to 0.137)	0.129 (−0.272 to 0.527)
Regularly visit MCHs	No	Ref.	Ref.
	Yes	−0.166 (−1.12 to 0.790)	0.155 (−0.45 to 0.765)
Educational status	Formal education	Ref.	Ref.
	Informal education	−3.29 (−4.34 to −2.24)***	−0.699 (−1.29 to −0.103)**
Household type	Owner	Ref.	Ref.
	Renter	2.056 (1.13 to 2.97)***	0.257 (−0.307 to 0.819)
	Relatives’ house	5.11 (4.06 to 6.17)***	0.937 (0.294 to 1.758)**
	IDP camps	1.01 (−0.097 to 2.127)	−0.247 (−0.970 to 0.476)
Household income (per month)	<$100	Ref.	Ref.
	$100–$199	0.70 (0.048 to 1.35)**	0.255 (−0.146 to 0.657)
	$200–$299	6.61 (5.255 to 7.97)***	1.525 (0.762 to 2.289)***
	>$300	10.004 (9.35 to 10.65)***	1.97 (0.693 to 3.255)**
Social support	No	Ref.	Ref.
	Yes	−.075 (−0.88 to 0.735)	0.127 (−0.336 to 0.592)
PHQ-9^a^			
GAD-7^b^		0.867 (0.818 to 0.917)***	0.724 (0.648 to 0.799)***
SOC^c^		−0.259 (−0.289 to −0.229)***	−0.097 (−0.125 to −0.068)***
Constant			6.708 (4.20 to 9.21)***
*R* ^2^			0.713

a Patient Health Questionnaire-9 (Depression);

b General Anxiety Disorder-7 scale (Anxiety);

c 13-item Sense of Coherence scale.

* *p* < .1.

** *p* < .05.

*** *p* < .01.

Living in rented housing was associated with a 2.06 higher depression score (*β* = 2.056, 95% CI: 1.13 to 2.97), and living with relatives was associated with a 5.11 higher score (*β* = 5.11, 95% CI: 4.06 to 6.17). Higher household income, $100–$199 (*β* = 0.70, 95% CI: 0.05 to 1.35), $200–$299 (*β* = 6.61, 95% CI: 5.26 to 7.97), and ≥ $300 per month (*β* = 10.0, 95% CI: 9.35 to 10.65), was associated with higher depression scores.

Anxiety symptoms also showed a strong positive association with depression (*β* = 0.86, 95% CI: 0.818 to 0.917), whereas a higher sense of coherence was associated with lower depression scores (*β* = −0.259, 95% CI: −0.289 to −0.229). Surprisingly, having no formal education was associated with lower depression scores (*β* = −3.29, 95% CI: −4.34 to −2.24). Other variables, such as pregnancy status, MCH visits, and social support, showed negative but non-significant associations.

In the multivariate model, only living with relatives (*β* = 0.937, 95% CI: 0.294 to 1.758), higher household income ($200–$299 and ≥$300), and greater anxiety symptoms (*β*= 0.724, 95% CI: 0.648 to 0.799) remained significantly associated with higher depression scores. Sense of coherence retained a protective effect.

### 3.3 Associations between anxiety symptoms (GAD-7), sense of coherence, social support, and sociodemographic factors

Univariate analyses for anxiety revealed a similar pattern (Table 3). Having more children (*β* = 0.202, 95% CI: 0.095 to 0.309) was associated with higher anxiety symptoms. Additionally, compared with participants living in their own homes, higher anxiety scores were observed among those renting (*β* = 2.023, 95% CI: 1.04 to 3.00), living with relatives (*β* = 4.67, 95% CI: 3.58–5.77), and living in IDP camps (*β* = 1.76, 95% CI: 0.56 to 2.96) were all associated with increased anxiety symptoms.

**Table 3:** Univariate and multivariate linear regression analyses for anxiety (GAD-7), SOC, social support, and demographic factors. (*n* = 899).

Variables	Categories	Univariate *β* (95% CI)	Multivariate *β* (95% CI)
Age		0.029 (−0.015 to 0.075)	−0.032 (−0.072 to 0.006)
Number of children		0.202 (0.095 to 0.309)***	0.072 (−0.017 to 0.162)
Marital status	Married	Ref.	Ref.
	Single	2.147 (0.608 to 3.68)	0.363 (−0.391 to 1.11)
Pregnancy status	Yes	Ref.	Ref.
	No	−0.674 (−1.35 to 0.0034)	−0.187 (−0.584 to 0.209)
Regularly visit MCHs	No	Ref.	Ref.
	Yes	−0.300 (−1.31 to 0.712)	−0.234 (−0.897 to 0.429)
Educational status	Formal education	Ref.	Ref.
	Informal education	−2.816 (−3.84 to −1.79)***	−0.165 (0.668 to 0.337)
Household type	Owner	Ref.	Ref.
	Renter	2.023 (1.04 to 3.00)***	0.345 (−0.346 to 1.036)
	Relatives’ house	4.67 (3.58 to 5.77)***	0.553 (−0.172 to 1.27)
	IDP camps	1.76 (0.562 to 2.96)***	0.992 (0.123 to 1.86)**
Household income	<$100	Ref.	Ref.
	$100–$199	0.269 (−0.339 to 0.879)	−0.231 (−0.621 to 0.16)
	$200–$299	6.19 (4.8 to 7.58)***	1.456 (0.704 to 2.207)***
	>$300	10.18 (9.23 to 11.12)***	3.065 (2.206 to 3.924)**
Social support	No	Ref.	Ref.
	Yes	−0.078 (−0.85 to 0.696)	−0.023 (−0.496 to 0.450)
PHQ-9^a^		0.775 (0.737 to 0.814)***	0.706 (0.66 to 0.753)***
GAD-7^b^			
SOC^c^		−0.21 (−0.244 to −0.184)***	−0.031 (−0.055 to −0.0062)***
Constant			3.803 (1.759 to 5.853)***
*R* ^2^			0.687

a Patient Health Questionnaire-9 (Depression);

b General Anxiety Disorder-7 scale (Anxiety);

c 13-item Sense of Coherence scale.

* *p* < .1.

** *p* < .05.

*** *p* < .01.

Higher income, $200–$299 (*β* = 6.19, 95% CI: 4.80 to 7.58) and ≥$300 per month (*β* = 10.18, 95% CI: 9.23 to 11.12), were respectively associated with increased anxiety symptoms. In contrast, a higher sense of coherence (*β* = −0.21, 95% CI: −0.244 to −0.184) and having no formal education (*β* = −2.816, 95% CI: −3.84 to −1.79) were associated with lower levels of anxiety symptoms.

After adjustment, living in refugee camps (*β* = 0.992, 95% CI: 0.123 to 1.86), higher income $200–$299 (*β* = 1.456, 95% CI: 0.704 to 2.207) and ≥$300 (*β* = 3.065, 95% CI: 2.206 to 3.924), and higher depression scores (*β* = 0.706, 95% CI: 0.660 to 0.753) remained positively associated with anxiety. Only the sense of coherence was negatively associated with anxiety (*β* = −0.031, 95% CI: −0.055 to −0.0062).

## 4 Discussions

This was the first study to investigate the prevalence of depressive and anxiety symptoms among Somali women living in Somalia and their association with SOC, perceived social support, and sociodemographic factors. Regarding settings featuring conflicts and crises, these contexts often result in significant psychological impacts, such as posttraumatic stress disorder (PTSD) and CMDs such as depression and anxiety (22). The study findings showed that the Somali context was not an exception, as the results showed that the prevalence of both depression and anxiety symptoms was high among Somali women living in Mogadishu. The results revealed that 65% of the study population had moderate to severe depression, while 51.3% had moderate to severe anxiety.

These findings align with the literature, such as the work of Hossaini et al. (21), who reported a high prevalence of depression and anxiety among Somali women in a Kenyan refugee camp, and the work of Ibrahim et al. (14), who reported that Somalia has consistently ranked among countries with the highest prevalence of CMDs. The aforementioned study further indicates that approximately one in every three Somali individuals has experienced some form of psychological disorder in their lifetime. These findings underscore the significant and persistent mental health burden within Somalia and among Somali women, as well as strengthen the assumption of a higher prevalence of CMDs in the Somali population. Moreover, this study and other studies, such as Tang et al. (23), describe a positive association between depression and anxiety, which can explain why the prevalences of these two disorders are similar. In addition, these findings help us understand how to provide mental health services effectively in the future.

Different factors were associated with CMDs among women in a relatively recent systematic study, such as poor education and being divorced, single, or widowed (1). Our study showed that the number of children, being single, and housing instability were linked with higher depression scores. The association between living with relatives and higher depressive symptoms may reflect hidden economic strain or reduced autonomy—everyday stressors in shared or dependent living arrangements in fragile settings. For anxiety, a similar pattern emerged, with housing conditions showing a strong relationship. In the multivariate model, women living in IDP camps had significantly higher anxiety scores than those in their own homes, which aligns with previous studies showing elevated anxiety among displaced Somali women (21). IDP environments typically involve overcrowding, insecurity, disrupted social networks, and inconsistent access to resources, all of which contribute to psychological distress (3, 24).

Interestingly, the study results showed that higher income was associated with higher depressive and anxiety symptoms, which may seem counterintuitive. A study conducted across 53 countries found that greater household spending, unlike material assets, was associated with higher odds of depression in adjusted analyses (25). Even though our study was not investigating household spending, it may be that higher income is associated with greater financial obligations to extended family or precarious income sources, leading to heightened stress. Significantly, several factors commonly linked to CMDs in other settings, such as age, marital status, pregnancy status, number of children, social support, and regular visits to MCH facilities, were not associated with depression or anxiety in the adjusted analyses. This differs from findings reported in other low-resource settings (1, 26). The lack of association in this study suggests that structural and environmental stressors in Somalia may overshadow the effects of individual demographic characteristics.

Another interesting finding was that having no formal education was associated with lower depressive symptoms. This contrasts with typical findings that link low education to worse mental health outcomes (1). However, a study conducted in Canada showed that individuals with lower educational attainment in specific subgroups, such as those not engaged in work, were found to have a lower likelihood of experiencing depression compared with those with higher education (27). We hypothesise that women with higher education may face greater responsibilities or higher expectations than those with less education, may report symptoms differently, or unmeasured factors may influence the relationship. This unexpected finding indicates a need for further research to understand how education interacts with cultural norms, stress exposure, and help-seeking behaviour among Somali women.

A consistent and theoretically meaningful finding was the protective role of SOC. Higher SOC was associated with lower symptoms of both depression and anxiety across models. This aligns with Antonovsky's theory and with empirical studies showing that SOC enhances coping and resilience in the face of adversity (10, 28, 29). However, despite relatively high SOC levels in the sample, many women still reported moderate to severe symptoms. This indicates that while SOC offers psychological protection, it may be insufficient to counterbalance the cumulative stressors associated with conflict, displacement, and economic instability.

### 4.1 Strengths and limitations

This study has several important strengths. It is among the few investigations to examine depressive and anxiety symptoms among Somali women in Mogadishu and draws on a relatively large sample distributed across nine districts, improving geographic representation. The use of standardized and previously validated measures adds methodological rigor, and the study contributes valuable evidence from a context where empirical mental health research remains limited.

Nonetheless, several limitations must be acknowledged. First, the study relied on self-reported questionnaires, which are inherently sensitive to recall issues, social desirability, and cultural nuances in symptom reporting. Because data collection took place in MCH facilities, some women may have intentionally overestimated their mental health concerns in hopes of accessing additional support, while others may have minimized symptoms due to mental health stigma. These opposing pressures complicate the interpretation of prevalence estimates.

Although the PHQ-9 and GAD-7 instruments have been used for Somali-speaking populations, the constructs they measure originate from Western psychiatric frameworks. Somali understandings of mental distress are often rooted in social, spiritual, or situational explanations rather than biomedical ones. Somalis have also been described as a people who use metaphor and poetry to convey emotional and social experiences. This rich expressive tradition may not translate seamlessly into the symptom-focused format of structured screening tools, potentially influencing how participants interpret and respond to individual items.

The use of convenience sampling in MCH clinics further limits generalizability, as women seeking maternal or child health services may differ from those who do not access such facilities. Additionally, the study was conducted during the COVID-19 pandemic, a period marked by heightened uncertainty and stress. It is therefore possible that the levels of depressive and anxiety symptoms reported here are elevated compared with what might have been observed prior to the pandemic. Moreover, women presenting to MCH clinics may already experience higher stress levels due to health concerns for themselves or their children, which could contribute to increased symptom reporting. Finally, given the study's cross-sectional design, causal interpretations cannot be made regarding the associations observed.

### 4.2 Conclusions

This study provides important insights into the mental health of Somali women living in Mogadishu, revealing that depressive and anxiety symptoms affect more than one-third of the population. These findings reflect broader patterns observed in conflict-affected settings and underscore the persistent mental burden facing women in environments marked by insecurity, displacement, and socioeconomic strain. Unlike prior studies highlighting factors such as marital status or social support, this study found limited associations with these variables after adjustment, suggesting that broader structural and contextual stressors may play a more influential role in shaping mental health outcomes.

Housing instability and displacement emerged as key factors. Living with relatives was associated with higher depression levels, while residing in IDP camps was linked with elevated anxiety symptoms. Higher income, unexpectedly, was also associated with increased symptoms of both depression and anxiety, emphasising the complexity of economic pressures in fragile settings. Additionally, while lower education is commonly identified as a risk factor for poor mental health, this study instead found an inverse association in some cases, indicating that educational attainment may influence mental health differently in this context.

Although a generally high level of sense of coherence (SOC) was observed, and SOC remained protective in all models, it did not prevent the notable levels of depressive and anxiety symptoms found. This suggests that internal coping resources alone are insufficient to offset the sustained external stressors faced by women in this post-conflict environment.

Taken together, these findings highlight the multifaceted nature of mental health challenges among Somali women and point to the need for context-sensitive, structural, and community-based interventions. Future research should explore additional determinants of mental health, including trauma exposure, household dynamics, and gender-specific stressors, and consider longitudinal designs to understand better how ongoing instability and systemic barriers shape mental health over time. Furthermore, given the cultural nuances in how mental distress is expressed and understood in Somali communities, future research should also prioritize culturally grounded assessment methods and qualitative approaches to complement standardized screening tools.

### 4.3 Relevance for clinical practice

This study highlights a critical need for integrated mental health care for Somali women, particularly in conflict-affected settings such as Mogadishu. The high prevalence of moderate to severe depression and anxiety among the study population underscores the urgent necessity for accessible, culturally appropriate, and sustainable mental health services.

First, primary health care and maternal and child health (MCH) centres should serve as key entry points for identifying and supporting women with common mental disorders (CMDs). Training health care workers at these facilities in basic mental health care, including screening, psychoeducation, and brief interventions, can bridge the existing service gap and reduce stigma associated with seeking mental health support.

Second, special attention must be given to women in internally displaced persons (IDP) camps, who exhibited significantly higher anxiety symptoms. Mental health interventions targeting this population should account for the trauma, instability, and insecurity these women face. Community-based psychosocial programs that address trauma, promote safety, and build resilience are essential in these settings.

Finally, the Somali government must take concrete steps to integrate and sustainably finance mental health services into the broader health system. This includes training healthcare professionals, investing in mental health infrastructure, and reducing reliance on international aid by developing local funding mechanisms.

## Data Availability

In accordance with ethical approval for the study (SNU/SPHR/005/2021), we are not authorized to make the data publicly available. All relevant data used for this paper are available upon request.
